# 

**Published:** 1887-06-04

**Authors:** 


### THE HOSPITAL

An Institution, Family, and Parochial Journal of Hospitals, Asylums, & all Agencies for the Care of the Sick, Criticism & News.

### THE HOSPITAL SATURDAY AND SUNDAY FUNDS SPECIAL NUMBER.

Contents.

"Let there be Light" - - - - - - 153

The Hospitals Week.—Objects and List of Meetings 154

Life in Hospital. By Our Special Commissioner.—Mrs. Johnson Argues the Point.—A Good Investment.—Can't it be done at Home?—The Pavilion Plan.—A Model Ward.—Sister Grace.—No Respect of Persons.—A Melancholy Night.—The Tight-rope Dancer.—Early Morning in the Ward.—The House Surgeon.—To Pay for your Keep.—Amateur Chaplains: Sunday Afternoon.—No Cakes Allowed.—The Operation.—Spray *v.* Germs: a Lilliputian Battle.—"He Prayeth Best who Loveth Best."—The Convalescent Home.—School in the Ward, etc., etc. - - - - - 155

The Sick and the Hospitals - - - - 170

A Year's Work in the Hospitals and Medical Charities of London - - - 171

Notices to the Clergy and Ministers of Religion - - - - - - 175

Hospital Sunday and the Queen's Jubilee - 175

The Origin of Hospitals - - - - 175

The Children's Corner.—Puzzles, Answers, etc. - 176

